# Visual deprivation modifies oscillatory activity in visual and auditory centers

**DOI:** 10.1080/19768354.2018.1474801

**Published:** 2018-05-17

**Authors:** Ping Pan, You Zhou, Fanghao Fang, Guannan Zhang, Yonghua Ji

**Affiliations:** Laboratory of Neuropharmacology and Neurotoxicology, Shanghai University, Shanghai, People's Republic of China

**Keywords:** Visual deprivation, cross-modal plasticity, oscillation, fiber projection

## Abstract

Loss of vision may enhance the capabilities of auditory perception, but the mechanisms mediating these changes remain elusive. Here, visual deprivation in rats resulted in altered oscillatory activities, which appeared to be the result of a common mechanism underlying neuronal assembly formation in visual and auditory centers. The power of high-frequency β and γ oscillations in V1 (the primary visual cortex) and β oscillations in the LGN (lateral geniculate nucleus) was increased after one week of visual deprivation. Meanwhile, the power of β oscillations in A1 (the primary auditory cortex) and the power of β and γ oscillations in the MGB (medial geniculate body) were also enhanced in the absence of visual input. Furthermore, nerve tracing revealed a bidirectional nerve fiber connection between V1 and A1 cortices, which might be involved in transmitting auditory information to the visual cortex, contributing to enhanced auditory perception after visual deprivation. These results may facilitate the better understanding of multisensory cross-modal plasticity.

## 1. Introduction

Blind individuals often display better tactile acuity (Van Boven et al. 2000; Goldreich and Kanics 2003), pitch discrimination (Gougoux et al. 2004) and odor identification (Cuevas et al. 2009; Beaulieu-Lefebvre et al. 2011; Zhou et al. 2017) than sighted individuals. In addition, early-blind subjects also demonstrate improved sound localization compared with sighted controls when attending to sounds in peripheral auditory space, whereas early-blind subjects perform similarly in the frontal field or even poorer than sighted subjects when the signal-to-noise ratio is low (Lessard et al. 1998; Roder et al. 1999; Zwiers et al. 2001). The central nervous system is able to adapt to the loss of one modality by undergoing plastic changes in its structural connectivity and neural interactions. Functional imaging studies of human and, recently, animal studies reveal that cross-modal plasticity might be attributed to reorganization of the deprived cortex to process the spared senses and adaptive plasticity of the spared cortices (Cohen et al. 1997; Sterr et al. 1998; He et al. 2012; Meng et al. 2015).

Dark-exposure visual deprivation in rodents leads to homeostatic up-regulation of excitatory synapses of layer 2/3 neurons in V1 (the primary visual cortex), increased miniature excitatory postsynaptic current (mEPSC) amplitude and up-regulated GluR1 expression, which returns to normal levels after re-exposure to light (Goel et al. 2006). Monocular enucleation of adult rodents initially reduces the activity in the deprived monocular zone of V1, while activity returns to normal levels after whisker stimulation (Van Brussel et al. 2011; Nys et al. 2014). These data provide an intriguing possibility that the deprived sensory cortex could be recruited to process remaining sensory information. The deprived sensory cortex is not the only part of the brain that adapts to the loss of its sensory inputs, but the spared sensory cortices also undergo synaptic changes. α-Amino-3-hydroxy-5-methyl-4-isoxazolepropionic acid (AMPA) receptor-mediated excitatory synaptic transmission in layer 2/3 of S1 (the primary somatosensory cortex) and A1 (primary auditory cortex) is reduced after dark-exposure (Goel et al. 2006; He et al. 2012). Moreover, loss of vision produces distinct neural circuit changes in the spared and deprived sensory cortices, shifting between feedforward and intracortical processing to allow adaptation (Petrus et al. 2015). In addition, the thalamocortical connections also undergo profound changes after the loss of sensory input. For example, visual deprivation strengthens thalamocortical synapses in A1 but not in V1, and deafening potentiates thalamocortical synapses in V1 but not in A1 (Petrus et al. 2014).

These findings demonstrate that adaptation of the spared and deprived sensory cortices may differ; however, little is known about the distinct underlying mechanisms. The goal of the present study was to investigate how neural oscillation and synchrony change in auditory and visual centers after the loss of visual input and whether there is direct neural connectivity between auditory and visual centers.

## 2. Material and methods

### 2.1. Animal models

Male Sprague–Dawley rats (including 60 rats at postnatal day 20 (P20) and 10 rats at P90) were obtained from Shanghai SLAC Animal Centre. All the animals were kept in the local animal facility and reared with *ad libitum* access to food and water. A visual deprivation model was created by exposing rats (P21) to complete darkness for 7 days (DE). Control animals were rats of a similar age and strain exposed to a normal visual experience with a 12-h light/dark cycle (the NR group) (Petrus et al. 2014; Meng et al. 2015). The investigation was approved by the Ethics Committee and the Committee of Animal Experimentation of Shanghai University. All efforts were made to minimize the number of animals used and their suffering.

### 2.2. LFP recording

Rats (P28) were anesthetized with chloral hydrate (10%, 4.5 mL/kg) and ethyl carbamate (20%, 2 mL/kg), placed in a stereotaxic frame and implanted with a 16-channel nickel-chromium microelectrode array (impedance less than 1 MΩ). Electrodes were accurately placed in layer 4 of A1 (6.3 mm posterior to bregma, 6.2 mm lateral to midline, 0.8 mm below the brain surface), layer 4 of V1 (5.2 mm posterior to bregma, 3.4 mm lateral to midline, 0.8 mm below the brain surface), medial geniculate body (MGB; 6.3 mm posterior to bregma, 3.4 mm lateral to midline, 4.9 mm below the brain surface), lateral geniculate nucleus (LGN; 5.2 mm posterior to bregma, 3.4 mm lateral to midline, 4.0 mm below the brain surface) according to brain topography. Recordings of the local field potential (LFP) were acquired in the absence of visual or acoustic stimuli in a dark and sound-attenuated cubicle (with background noise level at approximately 20 dB). To reduce various interferences of ambient electromagnetic fields, we placed the recording chamber in a Faraday cage. LFPs were acquired as broadband signals (0.1 Hz∼5 kHz) using a Plexon OmniPlex System (USA). Brains were sliced and stained with toluidine blue after recording to ensure that electrodes were located in the correct position, in each recording area of NR or DE group, six rats were successfully recorded.

### 2.3. Data analysis

The following data analysis steps were performed off-line with custom-written MATLAB scripts. After the data were imported into the MATLAB environment, a random 10-s epoch in each recording was selected and extracted to create a single file. LFP recordings were low-pass filtered with a cutoff at 300 Hz. Line noise artifacts were removed using a 50-Hz Butterworth notch filter. Power spectral density (PSD) was computed using the Welch technique, with Hamming windowing and a fast Fourier transform segment length of 512 samples with a 256-sample overlap. Changes in power were analyzed for five frequency oscillations (δ: ∼1–4 Hz, θ: ∼4–8 Hz, α: ∼8–13 Hz, β: ∼13–30 Hz, γ: ∼30–90 Hz). Wavelet packet decomposition was used to extract these five frequency bands. These oscillations were chosen because preliminary analyses showed that specific spectral changes occurred in these frequency bands when animals were anesthetized. The power of each oscillation was computed separately. All LFP energy data were expressed as the mean ± SEM. A two-tailed *t*-test was performed to compared differences across groups using a significance criterion of *p *< 0.05.

### 2.4. Neural tracing

Sprague–Dawley rats (at P20 and P90) were anesthetized with chloral hydrate (10%, 4.5 mL/kg) and ethyl carbamate (20%, 2 mL/kg). Alexa Fluor 488-conjugated cholera toxin subunit B (CTB, a retrograde tracer, Invitrogen) solution was injected into V1 (P20: 4.8 mm posterior to bregma, 2.5 mm lateral to midline, 0.7 mm below the brain surface. P90: 4.87 mm posterior to bregma, 3.9 mm lateral to midline, 1.2 mm below the brain surface) and A1 (P20: 4.9 mm posterior to bregma, 5.1 mm lateral to midline, 0.7 mm below the brain surface. P90: 4.7 mm posterior to bregma, 3.5 mm lateral to midline, 1.2 mm below brain surface) with a glass pipette at a flow rate of 0.2 µl/min. The sites at A1 and V1 both received 1 µl injections. After one day, the rats (P21) injected with CTB in A1 were randomly divided into the NR group (*n* = 3 rats) and DE group (*n* = 3 rats). The remaining rats (P21) injected with CTB in V1 were also randomly divided into the NR group (*n* = 3 rats) and DE group (*n* = 3 rats). The rats (P91) injected with CTB in A1 and V1 were reared under normal visual experience for 7 days (NR) (*n* = 10 rats). A schematic of the CTB injection is shown in a supplementary figure (Figure S1). Then, rats (P28 and P98) in deep anesthesia were perfused with sterile saline and 4% paraformaldehyde in 0.1 M phosphate buffer (pH = 7.4). Brain tissues were collected and placed in a 20% sucrose 0.1 M PB solution for dehydration until they sunk and were then moved into a 30% sucrose 0.1 M PB solution until the tissue sunk again. Frozen sections were cut 20 µm thick with a Cryostat Microtome (HM 525, Thermo Fisher). Confocal images of target areas in the injected side of the brain slice were acquired under a fluorescence microscope (H600L, Nikon).

## 3. Results

### 3.1. LFP oscillations in V1 and the LGN change after DE

**Figure 1.**
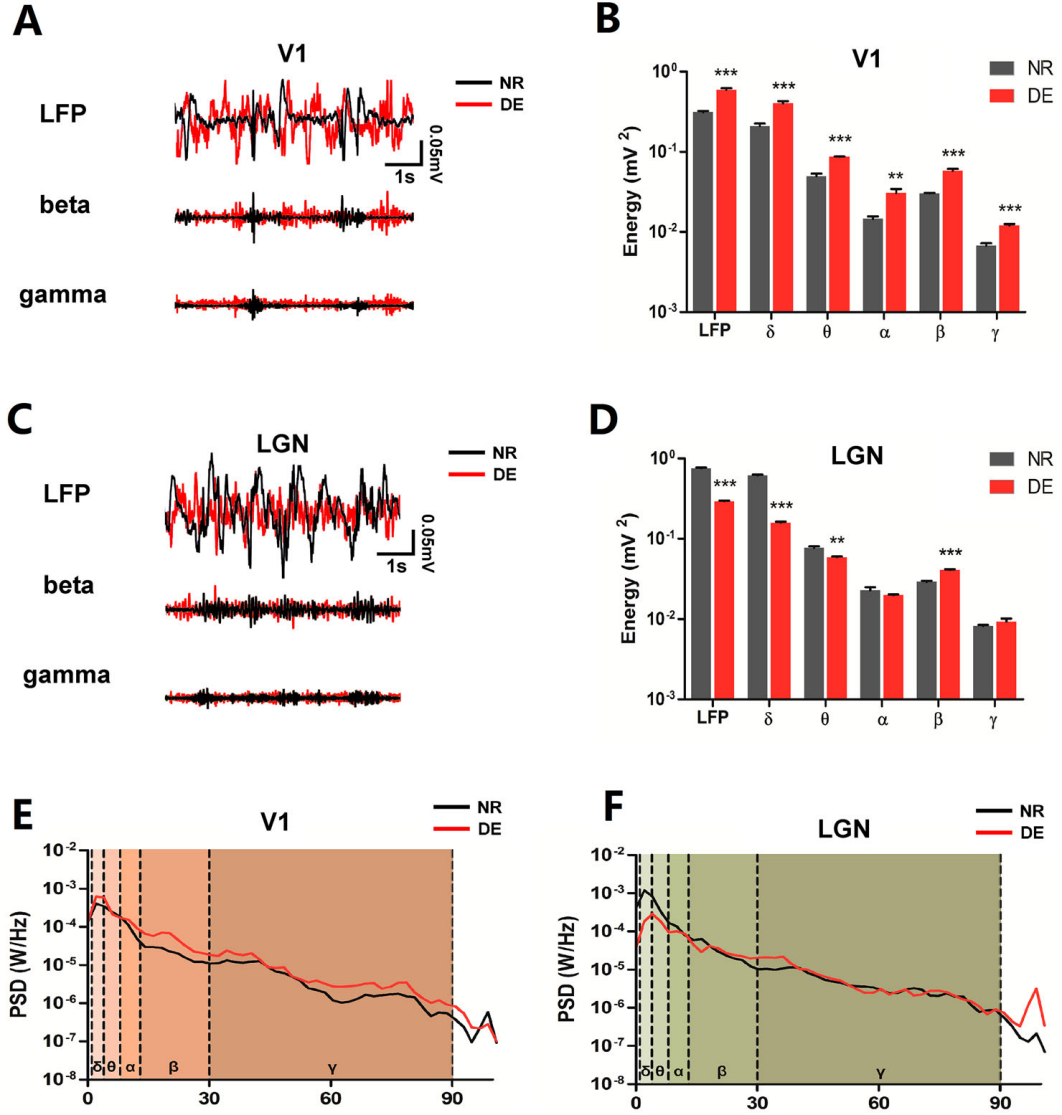
LFP characteristics of V1 and the LGN change after DE. (a) Random LFP segments from V1 of NR (shown with black line) and DE (shown with red line) rats alone and with β and γ oscillations extracted from these segments. (b) The power of five oscillations from V1 is shown in the bar graph (mV^2^), with data shown in a log scale. (c) Random LFP segments from the LGN of NR (shown with black line) and DE (shown with red line) rats alone and with β and γ oscillations extracted from these segments. (d) The power of five oscillations from the LGN is shown in the bar graph (mV^2^), with data shown in a log scale. (e) Average PSD from V1 of NR and DE rats is shown after it was normalized and computed with fast Fourier transform (FFT) (*n* = 4). (f) Average PSD from the LGN of NR and DE rats is shown after it was normalized and computed with FFT (*n* = 4). Each line chart was painted into five areas in order to distinguish one oscillation from the others. Data are shown as the mea*n* ± SEM. Asterisks indicate levels of significance by *t*-test with statistical significance at *P < 0.05, **P < 0.01 and ***P < 0.001.

*In vivo* LFP recordings showed that the raw LFP traces in visual thalamo-cortical centers were altered in the DE group (Figure 1(a,c)). After the raw traces were extracted into five frequency bands, the PSD was found to be embellished across different frequency bands after DE (Figure 1(e,f)). The total power of raw LFP oscillations in the V1 cortex was remarkably increased (increased by 89.97%, *p* < 0.001) in the DE compared to that in the NR group (NR, *n* = 6 rats; DE, *n* = 6 rats). The power of high-frequency β and γ oscillations was significantly enhanced after DE (increased by 92.36%, *p* < 0.001; increased by 79.1%, *p* < 0.001, respectively). The power of three low-frequency oscillations (δ, θ and α) in V1 were larger in the DE group than in the NR group (increased by 91.82%, *p* < 0.001; increased by 75.56%, *p* < 0.001; increased by 109.66%, *p* < 0.01, respectively) (Figure 1(b)). In addition, in contrast to the oscillations observed in V1, the total power of raw LFP oscillations in the LGN was obviously decreased following DE (decreased by 61.08%, *p* < 0.001) (NR, *n* = 6 rats; DE, *n* = 6 rats). The power of δ and θ oscillations was similarly decreased (decreased by 74.04%, *p* < 0.001; decreased by 23.28%, *p* < 0.01), whereas the β power was significantly increased after DE (increased by 41.03%, *p* < 0.001) (Figure 1(d)).

### 3.2. LFP oscillations in A1 and the MGB change after DE

**Figure 2.**
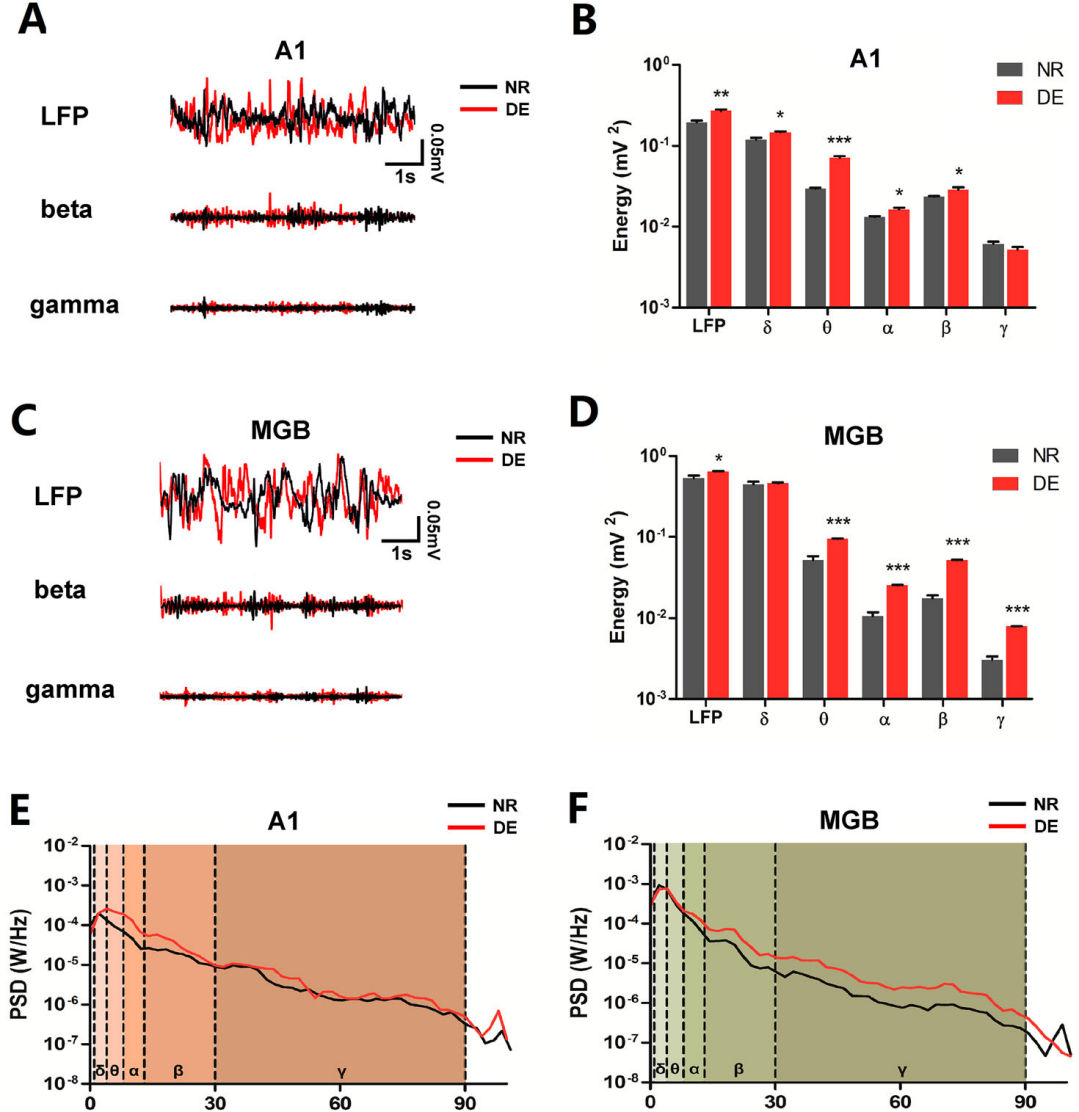
LFP characteristics of A1 and the MGB change after DE. (a) Random LFP segments from A1 of NR (shown with black line) and DE (shown with red line) rats alone and with β and γ oscillations extracted from these segments. (b) The power of five oscillations from A1 is shown in the bar graph (mV^2^), with data shown in a log scale. (c) Random LFP segments from the MGB of NR (shown with black line) and DE (shown with red line) rats alone and with β and γ oscillations extracted from these segments. (d) The power of five oscillations from the MGB is shown in the bar graph (mV^2^), with data shown in a log scale. (e) Average PSD from A1 is shown after it was normalized and computed with FFT (*n* = 4). (f) Average PSD from the MGB of NR and DE rats is shown after it was normalized and computed with FFT (*n* = 4). Each line chart was painted into five areas in order to distinguish one oscillation from the others. Data are shown as the mea*n* ± SEM. Asterisks indicate levels of significance by *t*-test with statistical significance at *P < 0.05, **P < 0.01 and ***P < 0.001.

Investigation of the adaptive changes in the auditory center revealed modifications of the raw LFP traces after DE (Figure 2(a,c)). Obvious changes in different frequency bands of the PSD were observed after DE (Figure 2(e,f)). The total power of raw LFP oscillations in A1 was increased after DE (increased by 37.63%, *p* < 0.01) (NR, *n* = 6 rats; DE, *n* = 6 rats). The power of high-frequency β oscillation in A1 was significantly enhanced in the DE group compared to that in the NR group (increased by 21.61%, *p* < 0.05). The power of three low-frequency oscillations (δ, θ and α) in A1 was larger in the DE group than in the NR group (increased by 23.52%, *p* < 0.05; increased by 141.84%, *p* < 0.001; increased by 23.48%, *p* < 0.05, respectively) (Figure 2(b)). Furthermore, in the MGB, the power of raw LFP oscillations was significantly larger in the DE group than in the NR group (increased by 21.07%, *p* < 0.05) (NR, *n* = 6 rats; DE, *n* = 6 rats), and the power of high-frequency β and γ oscillations was remarkably increased after DE (increased by 192.66%, *p* < 0.001; increased by 154.84%, *p* < 0.001). In addition, the remaining low-frequency oscillations (θ and α) were also enhanced after DE (increased by 83.37%, *p* < 0.001; increased by 137.74%, *p* < 0.001) (Figure 2(d)).

### 3.3. Thalamocortical and corticocortical connections in visual and auditory centers

**Figure 3.**
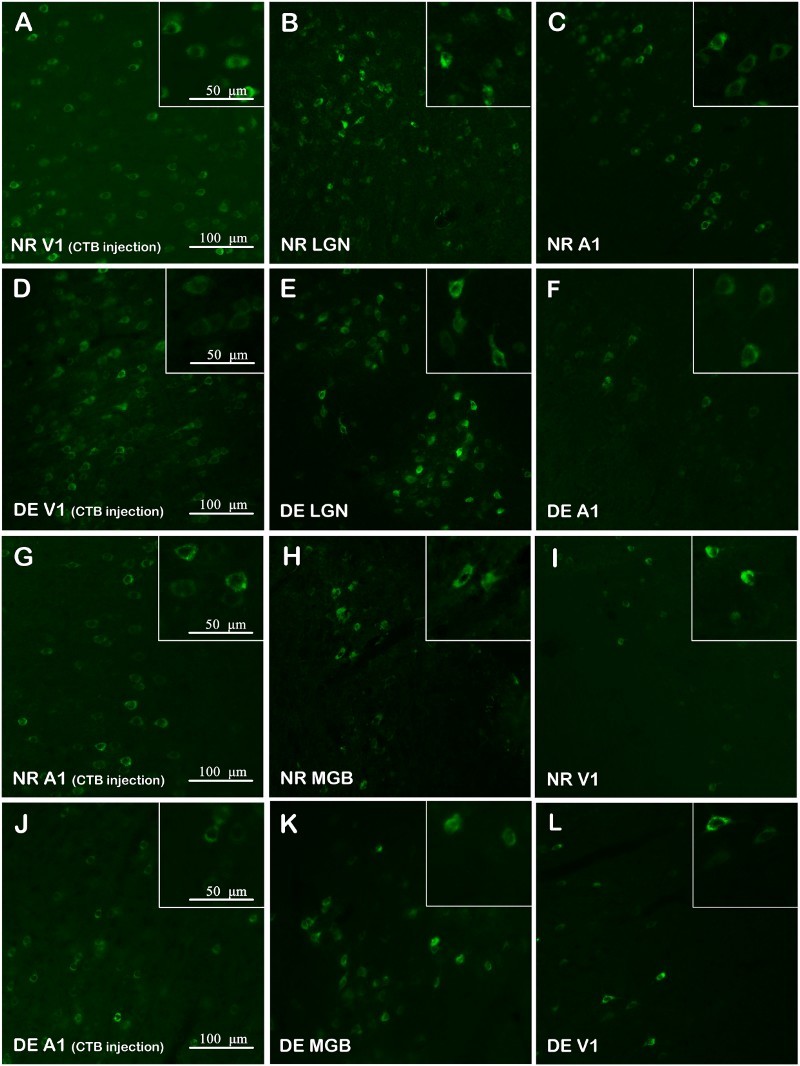
Retrograde tracing in visual and auditory centers of rats at P28. (a) The injection site (V1) of an NR rat at P28; CTB-labeled cells are shown in green. (b,c) Retrograde labeling of neurons in the LGN and A1 of an NR rat at P28 after CTB injection within V1. (d) The injection site (V1) of a DE rat at P28. (e,f) Retrograde labeling of neurons in the LGN and A1 of a DE rat at P28 after CTB injection within V1. (g) The injection site (A1) of an NR rat at P28. (h,i) Retrograde labeling of neurons in the MGB and V1 of an NR rat at P28 after CTB injection within A1. (j) The injection site (A1) of a DE rat at P28. (k,l) Retrograde labeling of neurons in the MGB and V1 of a DE rat at P28 after CTB injection within A1.

**Figure 4.**
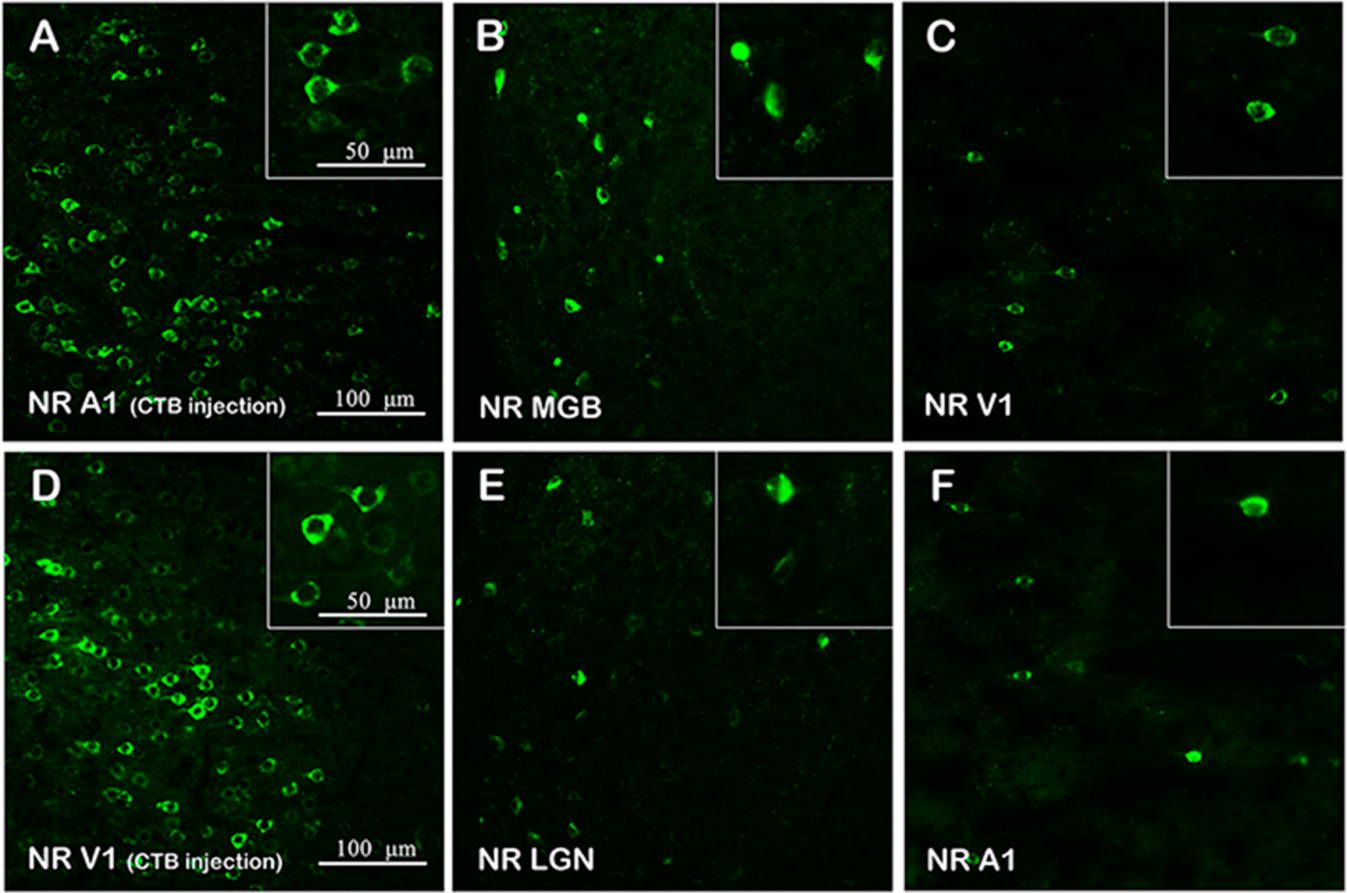
Retrograde tracing in visual and auditory centers of rats at P98. (a) The injection site (A1) of an NR rat at P98; CTB-labeled cells are shown in green. (b,c) Retrograde labeling of neurons in the MGB and V1 of a rat at P98 after CTB injection within A1. (d) The injection site (V1) of an NR rat at P98. (e,f) Retrograde labeling of neurons in the LGN and A1 of a rat at P98 after CTB injection within V1.

To examine patterns of corticocortical and thalamocortical connections in visual and auditory centers, we employed a retrograde tracer, CTB, to map the projections. The labeling of each group was performed and reproduced in no less than three animals (as shown in Figure S1); Figures 3 and 4 show example data from individual animals. Retrogradely labeled cells were found in the LGN and MGB of NR rats at P28 after CTB was injected into the V1 and A1 cortex, respectively (Figure 3(a,b,g,h)), demonstrating ascending fiber projections from the LGN to V1 cortex and MGB to A1 cortex. Likewise, CTB-labeled cells were also detected in the LGN and MGB of DE rats at P28 after injection within the V1 and A1 cortex (Figure 3(d,e,j,k)), indicating that the thalamocortical connections in the visual and auditory centers were unchanged after DE. Retrogradely labeled cells were also found in A1 and V1 of NR rats at P28 when CTB was injected into the V1 and A1 cortex, respectively (Figure 3(c,i)), indicating that fiber projections between V1 and A1 cortices were bidirectional. The bidirectional corticocortical connections were also shown in visual and auditory centers of DE rats at P28 (Figure 3(f,l)). Moreover, the same bidirectional corticocortical connections between V1 and A1 cortices were also detected in rats at P98 (Figure 4).

## 4. Discussion

### 4.1. LFP plasticity in visual and auditory centers after visual deprivation

Accumulating evidence highlights the often superior performance of auditory functions in blind individuals (Merabet et al. 2005; Pasqualotto and Proulx 2012). Although lacking visual input, the visual cortices of blind individuals are still active during the perception of the remaining sensory information (Merabet et al. 2005). LFP oscillations are often accompanied by synchronization of activity within a widespread cerebral area and deemed to have a common mechanism underlying neuronal assembly formation (David et al. 2009). In the current study, oscillations in the V1 cortex were found to undergo remarkable changes in the absence of visual input. The enhancement of raw LFP oscillations and high-frequency β and γ oscillations suggests that neural excitability in layer 4 of the V1 cortex was increased after DE. A previous study demonstrated that the mEPSCs in layer 2/3 pyramidal neurons in the V1 cortex were increased by visual deprivation, and this increment was reversed after the recovery of visual input (Goel et al. 2006). Together, these data indicate that cellular excitation of V1 is globally strengthened by visual deprivation and that the V1 cortex may be involved in processing other remaining senses when visual input is lacking. On the other hand, visual deprivation does not alter the strength of thalamocortical synapses to layer 4 of the V1 cortex (Petrus et al. 2014), hinting that neural activity in the LGN may remain unaltered after visual deprivation. Nevertheless, the present study demonstrated that LFP oscillations in the LGN were obviously embellished; the enhanced β oscillation and opposite plastic changes of other oscillations suggest that the LGN may be involved in cross-modal compensation after visual deprivation. The low-frequency rhythms (δ, θ and α oscillation) in visual centers also underwent changes after visual deprivation. However, as mentioned in a previous study, these changes were relatively easily influenced by anesthesia (Zhou et al. 2017). In addition, compared to the roles of high-frequency rhythms (Jia et al. 2013; Schmiedt et al. 2014; Brunet et al. 2015), the roles of low-frequency rhythms in cortices involved in sensory perception need to be further defined. For these reasons, the changes in the low-frequency rhythms observed in this study are not discussed in depth.

Loss of visual input leads to widespread compensatory plasticity across brain areas (Merabet et al. 2008). The strength of thalamocortical synapses to layer 4 of the A1 cortex was increased after a week visual deprivation (Petrus et al. 2014). In the current study, raw LFP oscillations as well as β oscillations in layer 4 of the A1 cortex were significantly heightened, potentially contributing to a cross-modal enhancement of auditory perception induced by a lack of visual input. Furthermore, the β and γ oscillations in the MGB were increased, which indicated that enhanced neural excitability and underlying factors may account for the potentiation of the thalamocortical projection to A1 during visual deprivation. The intracortical circuits in the auditory cortex may be altered by visual deprivation; the elaborate refinement of intra- and inter-laminar connections in the auditory cortex may be the basis of the cross-modal plasticity (Meng et al. 2015). Therefore, the enhanced LFP oscillations in A1 may be attributed to not only the increase in the strength of the thalamocortical projection but also the refinement of intracortical circuits in the A1 cortex. These results suggest that, in addition to the adaptation of the visual cortex to visual input deprivation, the auditory sensory cortices also undergo plastic changes. How does visual cortex involve in compensating and processing of auditory information, are there inherent connections between visual and auditory centers?

### 4.2. The corticocortical connections between the V1 and A1 cortices

Corticocortical connections and thalamocortical connections are involved in mediating auditory responses in the visual cortex of the blind (Klinge et al. 2010). Retrograde neural tracing in the current study verified innate ascending fiber projections of visual and auditory centers, namely, projections from the thalamic LGN to V1 cortex and from the thalamic MGB to A1 cortex. The thalamocortical synapses from MGB to A1 but not from LGN to V1 were strengthened after visual deprivation. In contrast, the thalamocortical synapses from LGN to V1 but not from MGB to A1 were enhanced after deafening (Petrus et al. 2014). Anterograde studies have found that the auditory cortex sends transient axons to visual areas (Dehay et al. 1988; Clarke and Innocenti 1990). In the current study, bidirectional corticocortical connections between the V1 and A1 cortices of rats were discovered, seemingly providing a direct and quick pathway for auditory information to be transmitted between the central visual and auditory system both with and without visual input. Functional magnetic resonance imaging has also provided clear evidence for stronger corticocortical connections from A1 to V1 in congenital blind humans than in sighted humans (Klinge et al. 2010). The peripheral visual representations of early visual areas V2 and prostriata project to the caudal auditory cortex in macaque monkeys, indicating that the projections between the caudal auditory cortex and low-level visual cortex show some level of reciprocity (Falchier et al. 2002). Moreover, the direct projections from the auditory cortex to V1 cortex and secondary visual area (V2) could also serve as a substrate for auditory influences over low-level visual processing (Falchier et al. 2010). Hence, the enhanced neural activity of the V1 cortex of vision-deprived rats in this study strongly hinted that the V1 cortex might be involved in receiving and processing auditory information and sending this information back to the A1 cortex through bidirectional corticocortical connectivity. In the present study, the corticocortical connectivity between the V1 and A1 cortices remained in the adult stage, further suggesting that the direct anatomical substrate might be involved in cross-modal plasticity. However, the complicated changes in the different oscillations likely imply that the LGN is involved in cross-modal compensation after visual deprivation. Certainly, several questions must be further investigated, such as whether the direct pathway linking the two primary cortices is modified during the loss of one sensory modality and whether there is a direct fiber connection between the thalamic LGN and MGB.

## 5. Conclusion

In summary, the present study demonstrated plastic changes in LFP oscillations in visual and auditory centers following visual deprivation as well as inherent bidirectional corticocortical connectivity between the V1 and A1 cortices, providing a novel perspective to further understand cross-modal plasticity.

## Supplementary Material

supplemental_figure.tif

## References

[CIT0001] Beaulieu-Lefebvre M, Schneider FC, Kupers R, Ptito M. 2011 Odor perception and odor awareness in congenital blindness. Brain Res Bull. 84:206–209. doi: 10.1016/j.brainresbull.2010.12.014 21236321

[CIT0002] Brunet N, Bosman CA, Roberts M, Oostenveld R, Womelsdorf T, De Weerd P, Fries P. 2015 Visual cortical gamma-band activity during free viewing of natural images. Cereb Cortex. 25:918–926. doi: 10.1093/cercor/bht280 24108806 PMC4379996

[CIT0003] Clarke S, Innocenti GM. 1990 Auditory neurons with transitory axons to visual areas form short permanent projections. Eur J Neurosci. 2:227–242. doi: 10.1111/j.1460-9568.1990.tb00415.x 12106050

[CIT0004] Cohen LG, Celnik P, Pascual-Leone A, Corwell B, Falz L, Dambrosia J, Honda M, Sadato N, Gerloff C, Catala MD, et al. 1997 Functional relevance of cross-modal plasticity in blind humans. Nature. 389:180–183. doi: 10.1038/38278 9296495

[CIT0005] Cuevas I, Plaza P, Rombaux P, De Volder AG, Renier L. 2009 Odour discrimination and identification are improved in early blindness. Neuropsychologia. 47:3079–3083. doi: 10.1016/j.neuropsychologia.2009.07.004 19616019

[CIT0006] David FO, Hugues E, Cenier T, Fourcaud-Trocme N, Buonviso N. 2009 Specific entrainment of mitral cells during gamma oscillation in the rat olfactory bulb. PLoS Comput Biol. 5(10):e1000551. doi: 10.1371/journal.pcbi.1000551 19876377 PMC2760751

[CIT0007] Dehay C, Kennedy H, Bullier J. 1988 Characterization of transient cortical projections from auditory, somatosensory, and motor cortices to visual areas 17, 18, and 19 in the kitten. J Comp Neurol. 272:68–89. doi: 10.1002/cne.902720106 2454978

[CIT0008] Falchier A, Clavagnier S, Barone P, Kennedy H. 2002 Anatomical evidence of multimodal integration in primate striate cortex. J Neurosci. 22:5749–5759. doi: 10.1523/JNEUROSCI.22-13-05749.2002 12097528 PMC6758216

[CIT0009] Falchier A, Schroeder CE, Hackett TA, Lakatos P, Nascimento-Silva S, Ulbert I, Karmos G, Smiley JF. 2010 Projection from visual areas V2 and prostriata to caudal auditory cortex in the monkey. Cereb Cortex. 20:1529–1538. doi: 10.1093/cercor/bhp213 19875677 PMC2882821

[CIT0010] Goel A, Jiang B, Xu LW, Song L, Kirkwood A, Lee HK. 2006 Cross-modal regulation of synaptic AMPA receptors in primary sensory cortices by visual experience. Nat Neurosci. 9:1001–1003. doi: 10.1038/nn1725 16819524 PMC1905492

[CIT0011] Goldreich D, Kanics IM. 2003 Tactile acuity is enhanced in blindness. J Neurosci. 23:3439–3445. English. doi: 10.1523/JNEUROSCI.23-08-03439.2003 12716952 PMC6742312

[CIT0012] Gougoux F, Lepore F, Lassonde M, Voss P, Zatorre RJ, Belin P. 2004 Neuropsychology: pitch discrimination in the early blind. Nature. 430:309. doi: 10.1038/430309a 15254527

[CIT0013] He K, Petrus E, Gammon N, Lee HK. 2012 Distinct sensory requirements for unimodal and cross-modal homeostatic synaptic plasticity. J Neurosci. 32:8469–8474. doi: 10.1523/JNEUROSCI.1424-12.2012 22723686 PMC3444293

[CIT0014] Jia X, Tanabe S, Kohn A. 2013 Gamma and the coordination of spiking activity in early visual cortex. Neuron. 77:762–774. doi: 10.1016/j.neuron.2012.12.036 23439127 PMC3632874

[CIT0015] Klinge C, Eippert F, Roder B, Buchel C. 2010 Corticocortical connections mediate primary visual cortex responses to auditory stimulation in the blind. J Neurosci. 30:12798–12805. doi: 10.1523/JNEUROSCI.2384-10.2010 20861384 PMC6633575

[CIT0016] Lessard N, Pare M, Lepore F, Lassonde W. 1998 Early-blind human subjects localize sound sources better than sighted subjects. Nature. 395:278–280. doi: 10.1038/26228 9751055

[CIT0017] Meng X, Kao JP, Lee HK, Kanold PO. 2015 Visual deprivation causes refinement of intracortical circuits in the auditory cortex. Cell Rep. 12:955–964. doi: 10.1016/j.celrep.2015.07.018 26235625 PMC4719125

[CIT0018] Merabet LB, Hamilton R, Schlaug G, Swisher JD, Kiriakopoulos ET, Pitskel NB, Kauffman T, Pascual-Leone A. 2008 Rapid and reversible recruitment of early visual cortex for touch. PloS one. 3(8):e3046. doi: 10.1371/journal.pone.0003046 18728773 PMC2516172

[CIT0019] Merabet LB, Rizzo JF, Amedi A, Somers DC, Pascual-Leone A. 2005 What blindness can tell us about seeing again: merging neuroplasticity and neuroprostheses. Nat Rev Neurosci. 6:71–77. doi: 10.1038/nrn1586 15611728

[CIT0020] Nys J, Aerts J, Ytebrouck E, Vreysen S, Laeremans A, Arckens L. 2014 The cross-modal aspect of mouse visual cortex plasticity induced by monocular enucleation Is Age dependent. J Comp Neurol. 522:950–970. doi: 10.1002/cne.23455 24037705

[CIT0021] Pasqualotto A, Proulx MJ. 2012 The role of visual experience for the neural basis of spatial cognition. Neurosci Biobeh Rev. 36:1179–1187. doi: 10.1016/j.neubiorev.2012.01.008 22330729

[CIT0022] Petrus E, Isaiah A, Jones AP, Li D, Wang H, Lee HK, Kanold PO. 2014 Crossmodal induction of thalamocortical potentiation leads to enhanced information processing in the auditory cortex. Neuron. 81:664–673. doi: 10.1016/j.neuron.2013.11.023 24507197 PMC4023256

[CIT0023] Petrus E, Rodriguez G, Patterson R, Connor B, Kanold PO, Lee HK. 2015 Vision loss shifts the balance of feedforward and intracortical circuits in opposite directions in mouse primary auditory and visual cortices. J Neurosci. 35:8790–8801. doi: 10.1523/JNEUROSCI.4975-14.2015 26063913 PMC4461685

[CIT0024] Roder B, Teder-Salejarvi W, Sterr A, Rosler F, Hillyard SA, Neville HJ. 1999 Improved auditory spatial tuning in blind humans. Nature. 400:162–166. doi: 10.1038/22106 10408442

[CIT0025] Schmiedt JT, Maier A, Fries P, Saunders RC, Leopold DA, Schmid MC. 2014 Beta oscillation dynamics in extrastriate cortex after removal of primary visual cortex. J Neurosci. 34:11857–11864. doi: 10.1523/JNEUROSCI.0509-14.2014 25164679 PMC4145181

[CIT0026] Sterr A, Muller MM, Elbert T, Rockstroh B, Pantev C, Taub E. 1998 Perceptual correlates of changes in cortical representation of fingers in blind multifinger braille readers. J Neurosci. 18:4417–4423. doi: 10.1523/JNEUROSCI.18-11-04417.1998 9592118 PMC6792812

[CIT0027] Van Boven RW, Hamilton RH, Kauffman T, Keenan JP, Pascual-Leone A. 2000 Tactile spatial resolution in blind braille readers. Neurology. 54:2230–2236. doi: 10.1212/WNL.54.12.2230 10881245

[CIT0028] Van Brussel L, Gerits A, Arckens L. 2011 Evidence for cross-modal plasticity in adult mouse visual cortex following monocular enucleation. Cereb Cortex. 21:2133–2146. doi: 10.1093/cercor/bhq286 21310780

[CIT0029] Zhou Y, Fang FH, Pan P, Liu ZR, Ji YH. 2017 Visual deprivation induce cross-modal enhancement of olfactory perception. Biochem Biophys Res Commun. 486:833–838. doi: 10.1016/j.bbrc.2017.03.140 28359762

[CIT0030] Zwiers MP, Van Opstal AJ, Cruysberg JR. 2001 A spatial hearing deficit in early-blind humans. J Neurosci. 21(RC142):141–145. 10.1523/JNEUROSCI.21-09-j0002.2001 PMC6762556 11312316

