# Supplementary figures and images for: Visual deprivation modifies oscillatory activity in visual and auditory centers
Source: Anim Cells Syst (Seoul). 2018 May 17;22(3):149–56. doi: 10.1080/19768354.2018.1474801 (PMC6138323; doi:10.1080/19768354.2018.1474801)

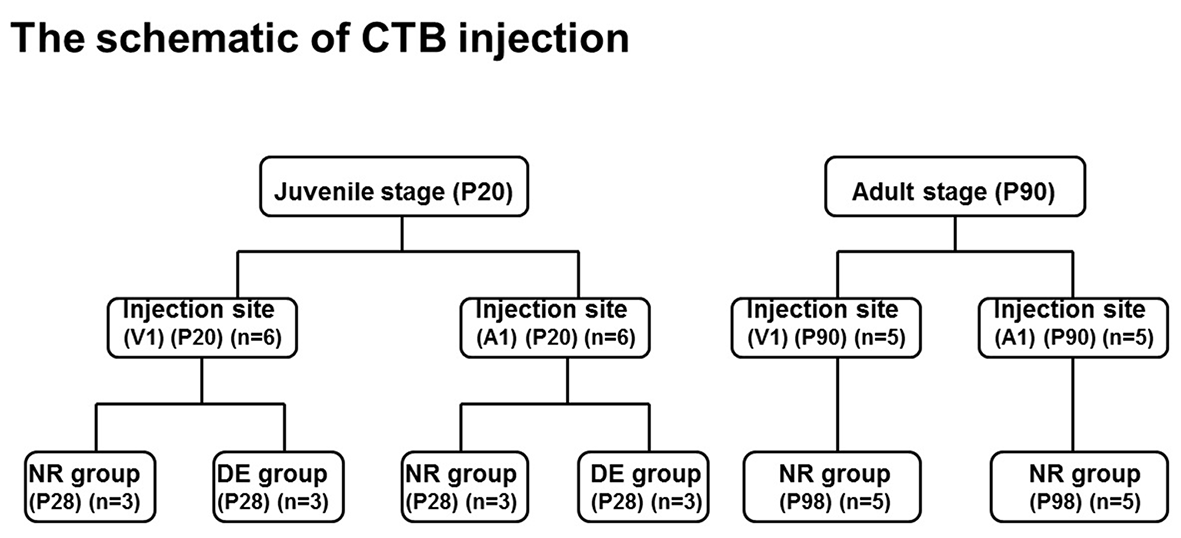

Supplement: supplemental_figure.tif [file TACS_A_1474801_SM0051.tif]
